# Solubility of palbociclib in supercritical carbon dioxide from experimental measurement and Peng–Robinson equation of state

**DOI:** 10.1038/s41598-023-29228-1

**Published:** 2023-02-07

**Authors:** Gholamhossein Sodeifian, Chieh-Ming Hsieh, Amirmuhammad Tabibzadeh, Hsu-Chen Wang, Maryam Arbab Nooshabadi

**Affiliations:** 1 Department of Chemical Engineering, Faculty of Engineering, University of Kashan, Kashan, 87317-53153 Iran; 2 Laboratory of Supercritical Fluids and Nanotechnology, University of Kashan, Kashan, 87317-53153 Iran; 3 Modeling and Simulation Centre, Faculty of Engineering, University of Kashan, Kashan, 87317-53153 Iran; 4 Department of Chemical and Materials Engineering, National Central University, Taoyuan, 320317 Taiwan; 5 Bolvar Ghotbe Ravandi, Islamic Azad University of Kashan, Ostaadan Street, Kashan, 87159-98151 Iran

**Keywords:** Chemical engineering, Chemical engineering

## Abstract

Palbociclib is a poorly water-soluble medicine which acts against metastatic breast cancer cells. Among various techniques to improve the solubility of this medicine, applying supercritical technologies to produce micro- and nano-sized particles is a possible option. For this purpose, extraction of solubility data is required. In this research, the solubility of palbociclib in supercritical carbon dioxide (ScCO_2_) at different equilibrium conditions was measured at temperatures between 308 and 338 K and pressures within 12–27 MPa, for the first time. The minimum and maximum solubility data were found to be 8.1 × 10^–7^ (at 338 K and 12 MPa) and 2.03 × 10^–5^ (at 338 K and 27 MPa), respectively. Thereafter, two sets of models, including ten semi-empirical equations and three Peng–Robinson (PR) based integrated models were used to correlate the experimental solubility data. Bian’s model and PR equation of state using van der Waals mixing rules (PR + vdW) showed better accuracy among the examined semi-empirical and integrated models, respectively. Furthermore, the self-consistency of the obtained data was confirmed using two distinct semi-empirical models. At last, the total and vaporization enthalpies of palbociclib solubility in ScCO_2_ were calculated from correlation results of semi-empirical equations and estimated to be 40.41 and 52.67 kJ/mol, respectively.

## Introduction

A review of the cancer statistics shows that breast cancer is the most commonly diagnosed among women worldwide, with an estimated 2.3 million cases and more than a quota of 11% of all cancer cases, in 2020^1^. Considering this fact, improved access to treatment is one of the main solutions against rising incidence of cancer related deaths. Among various types of cancer treatment, targeted drug therapy is one of the most common biological techniques, i.e., the small-molecule drugs such as cancer growth inhibitor can help to find and target specific parts of cancer cells. Because of low molecular weights, small molecules can penetrate the cell membrane easily to affect protein molecules, such as cyclin-dependent kinase 4 and 6 (or CDK 4 and CDK 6, respectively). Although these proteins play the primary roles in cell cycle progression, their increased activity causes loss of cell cycle control and fast cellular dividing. By blocking these proteins and their major enzymatic actions, the above mentioned drugs aim to put a brake on the spreading of cancer cells^2^. In this regard, palbociclib is a highly selective inhibitor against the proliferation of breast cancer cells, capable of targeting the proteins that stimulate cellular growth and multiplying.

Based on the biopharmaceutics classification system (BCS), the solubility and permeability of drugs are the major factors required for the description of oral drug absorption^3^. With respect to this fact, improving drug solubility in water, especially for lipophilic drugs, is one of the critical challenges in the pharmaceutical industry. In this regard, particle size manipulation can be helpful in meeting the challenge. For this purpose, the generation of new solubility data on palbociclib, especially in supercritical carbon dioxide (ScCO_2_), can clear the way for micro and nanoparticle production via relevant processes such as rapid expansion of supercritical solution (RESS)^4^ and supercritical anti-solvent precipitation (SAS)^5^. The latter is based on fast injection of liquid solution of organic/inorganic solvent and solid solute into the supercritical fluid (SCF), while the former is carried out by depressurization the mixture of supercritical fluid and solid solute through a heated nozzle at high speed. Moreover, the knowledge of solid solubility values as well as its behavior versus temperature and pressure is of high value for optimizing not only particle size but also yield and morphology of the product^6^.

When talking about CO_2_, topics such as greenhouse gases, global warming and the relevant environmental considerations maybe occur to someone. However, the magic effects of its supercritical state on the development of numerous fields are undeniable, including extraction of organic compounds^7–11^, drug solubility measurement^12–17^, particle production^18–20^, power generation^21^, polymer synthesis and processing^22,23^, impregnation process^24^, tissue engineering^25^ and food industry^26^. Moreover, low toxicity, safety, low environmental impacts and accessible critical conditions make CO_2_ a special and the most frequently used supercritical solvent, both on the laboratory scale and in the industrial level processes.

To obtain the solubility of solid substances in ScCO_2_ within a wide range of temperature and pressure, substantial numbers of correlative and predictive tools have been proposed during recent decades^27^, which are primarily classified into three categories including equations of state^28–30^, empirical and semi-empirical models^12,31^ and artificial intelligence models^32–34^. This study measured palbociclib solubility in ScCO_2_ and correlated the obtained data set by ten semi-empirical models and three integrated Peng–Robinson (PR) equation of state (EoS) based models along with evaluating the accuracy of each approach.

## Experiment

### Materials

Palbociclib in the form of powder with a purity exceeding 99.0% was purchased from Parsian Pharmaceutical Company (Tehran, Iran). Further information about the molecular structure along with thermodynamic properties of palbociclib are presented in Table 1. CO_2_ with a purity of 99% was supplied by Fadak Company (Kashan, Iran). Dimethyl sulfoxide (DMSO) produced by Merck (Darmstadt, Germany) with a minimum purity of 99.9% was used as trapping solvent after the solubility process.

**Table 1:** Thermodynamic properties and molecular structure of palbociclib.

	Palbociclib
Molecular Structure	
Formula	C_24_H_29_N_7_O_2_
CAS number	571190-30-2
*λ*_max_ (nm)	265
*T*_m_ (K)^68^	545.19
Δ $\overline{H }$_m_ (kJ∙mol^−1^)^68^	23.7
*T*_c_ (K)^a^	1254.31
*P*_c_ (MPa)^a^	1.6069
$\omega$ (–)^a^	0.4660
${\overline{V} }^{S}$(cm^3^ mol^−1^)^b^	303.85

^a^The values of *T*_c_ and *P*_c_ were estimated by PR + COSMOSAC EoS^70,71^ and then rescaled by a factor of 0.88. The rescale factor is determined from regression of experimental solubility data together with *k*_*ij*_^78,79^. The Lee-Kesler equation^72^ was used to estimate the acentric factor.

^b^The molecular cavity volume in the COSMO solvation calculation was used as the solid molar volume.

### Experimental apparatus and procedure

**Figure 1:**
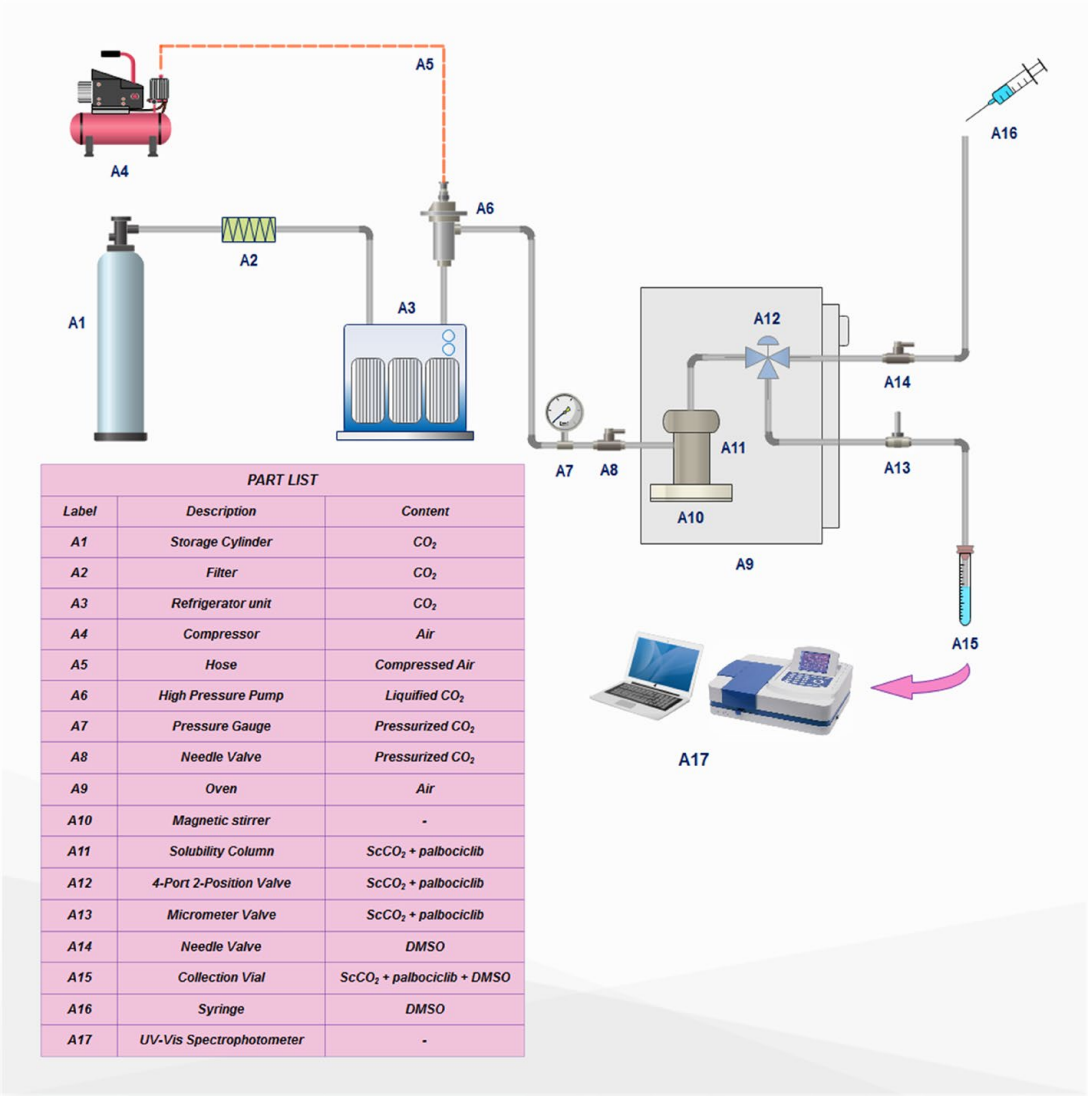
Experimental setup for supercritical solubility measurement.

The apparatus utilized in this work is depicted in Fig. 1 in which CO_2_ storage cylinder, filter (Hylok, 6000 psi), refrigerator unit, air compressor (Ronix, RC 5010), high pressure pump (Haskel pump, MSHP-110), oven (Froilabo AE-60, France) and solubility column composed the major parts of the supercritical unit. 500 mg powder of the sample along with 2 mm-diameter glass beads were loaded to the solubility column with a capacity of 70 ml. Then the column was placed in the oven. By starting the process, CO_2_ flowed through a molecular sieve filter with pore sizes of 1 μm from the storage cylinder into the refrigerator, where CO_2_ was liquefied by a reduction of temperature to 258 K. Then, liquid CO_2_ was entered into the reciprocating pump to reach the desired pressure of the supercritical fluid state. Downstream of the pump, a pressure gauge (WIKA, Germany) with an accuracy of ± 1 bar was installed for measuring and visual observing the supercritical CO_2_ pressure. Thereafter, by opening the needle valve of column input, ScCO_2_ was passed and brought into direct contact with the powder sample. To keep and control the desired temperature of the column, the oven was equipped with a platinum resistance thermometer (Pt 100 temperature sensor) along with a temperature monitoring system with an accuracy of 0.1 K. In this study, the time required for attaining equilibrium was set to 60 min. After equilibrium, 600 μl of saturated ScCO_2_ sample was discharged via a two-state four-port valve into a vial preloaded with a given volume of DMSO.

To determine the concentration of palbociclib in each solution sample corresponding to a particular pressure and temperature, a UV–Vis spectrophotometer (Cintra 101, GBC Scientific Equipment Ltd.) was employed along with a set of standard solutions. An analytical balance (LS-220 A SCS, Precisa, Swiss) with an accuracy of 0.0001 g was applied for mass measurement and standard solution preparation. The calibration curve (with a regression coefficient of about 99%) was established by analyzing concentrations of standard solutions with the mentioned UV–Vis spectrophotometer at the wavelength of maximum absorbance (λmax) of 265 nm. Subsequently, the equilibrium solubility (*S*) in terms of grams per liter and the equilibrium mole fraction (*y*) were obtained by the following relationships:

1 $${S}_{2}=\frac{{C}_{2}\times {V}_{s}}{{V}_{l}}$$

2 $${y}_{2}=\frac{{n}_{2}}{{n}_{1}+{n}_{2}}$$

3 $${n}_{1}=\frac{{\rho }_{1}\times {V}_{l}}{{M}_{1}}$$

4 $${n}_{2}=\frac{{C}_{2}\times {V}_{s}}{{M}_{2}}$$

where *n* and *M* represent the moles number and molecular mass of components in the sampling loop, respectively. In this binary system, subscripts 1 and 2 stand for ScCO_2_ and palbociclib as solvent and solute, respectively. *C*_2_ as the concentration of palbociclib (in terms of grams per liter) solved in each vial was obtained from the calibration curve. ${V}_{s}$ and ${V}_{l}$ refer to the volume, in terms of liter, of the solution and sampling loop, respectively. ${\rho }_{1}$ is ScCO_2_ density obtained from the NIST database at the corresponding conditions of temperature and pressure.

Reliability of the experimental apparatus and procedure was previously examined by measuring the solubility of two well-known substances, namely Alpha-tocopherol and naphthalene at different operational conditions and comparing the obtained results with the data reported in the literature^35^. In recent years, Sodeifian and coworkers utilized the apparatus frequently for various research purposes related to supercritical fluids processes, so more detailed experimental procedures are available in their published papers^23,36–39^.

## Results and discussion

### Experimental palbociclib solubility data

**Figure 2:**
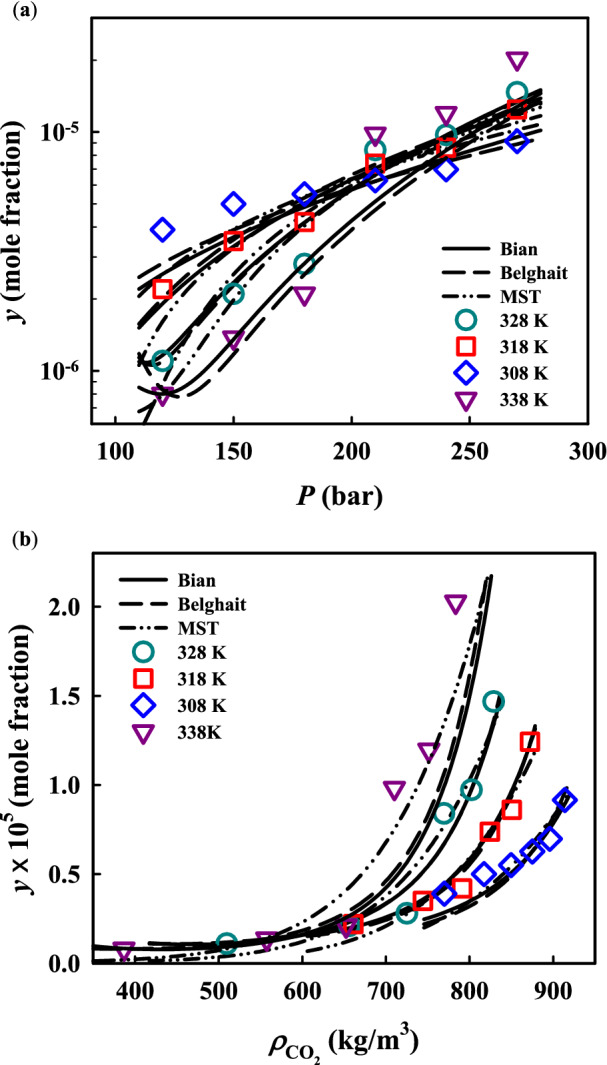
Palbociclib solubility in ScCO_2_ versus (**a**) pressure and (**b**) density from experimentation and three semi-empirical models.

According to what was mentioned above, solubility values of palbociclib in ScCO_2_ were obtained at planned pressures and temperatures, containing a set of 72 experimental data (24 data as primaries and 48 data as replications). These data, including equilibrium solubility (*S*) and equilibrium mole fraction (*y*), are presented in Table 2. It should be noted that each reported solubility datum is the average of three distinct experimental runs. As can be found in Table 2 and the corresponding data points presented in different shapes and colors on both Figs. 2a and 4, solubility values increase by increasing pressure along each isotherm. The reason is often attributed to the increment of CO_2_ solvating power due to increasing density under higher pressures. Consequently, more intensive molecular interaction between ScCO_2_ and solute particles is followed by more solubility of palbociclib. However, the influence of temperature is dualistic. Although the solubility values are adversely affected by temperature elevation at and under the pressure of 18 MPa, a positive relationship between solubility and temperature is observed at and above the pressure of 21 MPa. Therefore, it is concluded that there is a crossover point between the range of 18 and 21 MPa for palbociclib solubility in ScCO_2_ in which the isotherm curves intersect each other. As demonstrated by Figs. 2a and 4, higher compactness of experimental values at the right endpoint of this range leads to conclusion that the pressure crossover point must be in the vicinity of 21 MPa. Due to playing two simultaneous different roles at pressures under and above the crossover range, the effects of temperature rising on the solubility values are not as simple as that of pressure to justify. In this regard, temperature elevation makes ScCO_2_ density less, resulting in decreasing the solvating power. On the other side, solute’s vapor pressure followed by solubility would be raised, affected by temperature elevation. Depending upon which factor prevails, the type of relationship between solubility and temperature is predictable. In this study, the dominant factor under and above the crossover range is the ScCO_2_ density and palbociclib’s vapor pressure, respectively. These results are similar to those reported in other investigations on measuring the solubility of lansoprazole^40^, salsalate^41^, chloroquine^42^, glibenclamide^43^ and haloperidol^44^.

**Table 2:** Experimental data of palbociclib solubility in ScCO_2_.

*T* (K)	*P* (MPa)	${\rho }_{{\mathrm{CO}}_{2}}$^a^ (kg∙m^−3^)	*y* × 10^5^ (mole fraction)	*Std* (*y*) × 10^5^ (mole fraction)^b^	*U*($\overline{y }$) × 10^5^ (mole fraction)^c^	Solubility (g∙L^−1^)
308	12.0	769	0.391	0.012	0.027	0.0305
	15.0	817	0.501	0.024	0.053	0.0415
	18.0	849	0.552	0.021	0.047	0.0475
	21.0	875	0.627	0.011	0.034	0.0557
	24.0	896	0.697	0.012	0.036	0.0635
	27.0	914	0.914	0.021	0.056	0.0849
318	12.0	661	0.221	0.010	0.022	0.148
	15.0	744	0.349	0.015	0.033	0.264
	18.0	791	0.419	0.012	0.030	0.337
	21.0	824	0.738	0.011	0.038	0.618
	24.0	851	0.860	0.013	0.043	0.743
	27.0	872	1.242	0.041	0.096	0.1101
328	12.0	509	0.110	0.004	0.009	0.0057
	15.0	656	0.210	0.004	0.012	0.0140
	18.0	725	0.281	0.013	0.030	0.0206
	21.0	769	0.840	0.019	0.053	0.0656
	24.0	802	0.972	0.021	0.059	0.0792
	27.0	829	1.468	0.032	0.091	0.1235
338	12.0	388	0.081	0.003	0.007	0.0031
	15.0	557	0.137	0.002	0.007	0.0078
	18.0	652	0.211	0.003	0.011	0.0139
	21.0	710	0.981	0.041	0.093	0.0708
	24.0	751	1.197	0.011	0.057	0.0914
	27.0	783	2.027	0.091	0.202	0.1613

*Standard uncertainty values (*u*) for temperature and pressure are *u*(*T*) = 0.1 K and *u*(*P*) = 0.1 MPa.

^a^CO_2_ density was obtained from national institute of science and technology (NIST) chemistry webbook (http://webbook.nist.gov/chemistry/).

^b^Experimental standard deviation was obtained by $Std\left({y}_{i}\right)=\sqrt{\sum_{j}({y}_{j}-\overline{y })/n-1}$.

^c^Expanded uncertainty was determined from $U(\overline{y })=k\cdot {u}_{\mathrm{comb}}$ with the coverage factor *k* = 2 (the level of confidence of approximately 95%) and the relative combined standard uncertainty $u_{{comb}} /\bar{y} = \sqrt {\mathop \sum \limits_{i} \left( {P_{i} \cdot u_{i} \left( {x_{i} } \right)/x_{i} } \right)^{2} }$.

### Solubility correlation with semi-empirical models

The experimental palbociclib solubility in ScCO_2_ was correlated by ten semi-empirical models. Table 3 summarizes the equations and abbreviations of these ten semi-empirical models. The objective function of the average absolute relative deviation in percentage (AARD%) was used for *N* palbociclib solubility data from experiments (*y*^exp^) and semi-empirical models (*y*^cal^) to obtain adjustable parameter values of these semi-empirical models:

**Table 3:** Semi-empirical models.

Model	Equations
Chrastil^48^	$\mathrm{ln}{S}_{i}={a}_{0}\mathrm{ln}{\rho }_{{\mathrm{CO}}_{2}}+\frac{{a}_{1}}{T}+{a}_{2}$
MST^47^	$T\mathrm{ln}\left({y}_{i}\mathrm{P}\right)={a}_{0}+{a}_{1}{\rho }_{{\mathrm{CO}}_{2}}+{a}_{2}T$
K-J^73^	$\mathrm{ln}{y}_{i}={a}_{0}+{a}_{1}{\rho }_{{\mathrm{CO}}_{2}}+\frac{{a}_{2}}{T}$
Bartle^49^	$\mathrm{ln}\left({y}_{i}P/{P}_{\mathrm{ref}}\right)={a}_{0}+{a}_{1}({\rho }_{{\mathrm{CO}}_{2}}-{\rho }_{\mathrm{ref}})+\frac{{a}_{2}}{T}$
Bian^74^	$\mathrm{ln}{y}_{i}={a}_{0}+\frac{{a}_{1}}{T}+\frac{{a}_{2}\rho }{T}+({a}_{3}+{a}_{4}\rho )\mathrm{ln}\rho$
Garlapati^75^	$\mathrm{ln}{y}_{i}={a}_{0}+({a}_{1}+{a}_{2}\rho )\mathrm{ln}\rho +\frac{{a}_{3}}{T}+{a}_{4}\mathrm{ln}(\rho T)$
Keshmiri^76^	$\mathrm{ln}{y}_{i}={a}_{0}+\frac{{a}_{1}}{T}+{a}_{2}{P}^{2}+({a}_{3}+\frac{{a}_{4}}{T})\mathrm{ln}\rho$
Khansary^77^	$\mathrm{ln}{y}_{i}=\frac{{a}_{0}}{T}+{a}_{1}P+\frac{{a}_{2}{P}^{2}}{T}+\left({a}_{3}+{a}_{4}P\right)\mathrm{ln}\rho$
Sodeifian^12^	$\mathrm{ln}{y}_{i}={a}_{0}+{a}_{1}\frac{{P}^{2}}{T}+{a}_{2}\mathrm{ln}\left(\rho T\right)+{a}_{3}\left(\rho \mathrm{ln}\rho \right)+{a}_{4}P\mathrm{ln}T+{a}_{5}\frac{\mathrm{ln}\rho }{T}$
Belghait^31^	$\mathrm{ln}{y}_{i}={a}_{0}+{a}_{1}\rho +{a}_{2}{\rho }^{2}+{a}_{3}\rho T+{a}_{4}T+{a}_{5}{T}^{2}+{a}_{6}\mathrm{ln}\rho +\frac{{a}_{7}}{T}$

*S_*i*_: solubility in kg∙m^−3^,* y*_*i*_: solubility in mole fraction,* T*: temperature in K, *ρ*: ScCO_2_ density in kg∙m^−3^,* P*: pressure in bar, *a*_0_ ~ *a*_7_: adjustable model parameters, *P*_ref_: reference pressure (= 1 bar), *ρ*_ref_: reference density (= 700 kg∙m^−3^).

5 $$\mathrm{AARD\%}=\frac{1}{N}\sum_{i=1}^{N}\frac{\left|{y}_{i}^{\mathrm{exp}}-{y}_{i}^{\mathrm{cal}}\right|}{{y}_{i}^{\mathrm{exp}}}\times 100\%$$

Table 4 lists the optimal adjustable parameter values of each semi-empirical model along with the AARD% from these models using the optimized parameters. Bian’s and Belghait’s model had the highest accuracy with the lowest AARD% of 15.06% and 16.21%, respectively, while the other models gave similar AARD% ranging from 23.10 to 25.94%. Figure 2 compares the correlation results from Bian’s, Belghait’s and MST models and experimental solubility. As can be seen, the correlation results are generally consistent with the experimental data. Both Bian’s and Belghait’s models can provide lower overall deviations and showed a wide pressure range (16 ~ 27 MPa) for the crossover region. Although the MST model gave a slightly higher deviation, its results showed a narrow pressure range (around 21 MPa) for the crossover region, which was consistent with the experimental observation. Such a difference on describing the crossover region may be attributed to the function form and considered independent variables of the semi-emiprical model. A wide range of the crossover region makes Bian’s and Belghait’s models better for describing palbociclib solubility behavior at lower pressures and giving lower overall deviations.

**Table 4:** Parameter values in semi-empirical models.

Model	Parameters								AARD%
	${a}_{0}$	${a}_{1}$	${a}_{2}$	${a}_{3}$	${a}_{4}$	${a}_{5}$	${a}_{6}$	${a}_{7}$	
Chrastil	7.5314	−4860.461	−38.0736	–	–	–	–	–	24.51%
MST	−11,783.097	4.4410	19.0791	–	–	–	–	–	24.20%
K–J	0.4908	0.0108	−6767.2317	–	–	–	–	–	23.73%
Bartle	11.9754	0.0117	−6335.4073	–	–	–	–	–	24.20%
Bian	−20.3979	1.42 $\times$ 10^4^	−24.0799	−6.0178	0.01206	–	–	–	15.60%
Garlapati	−242.7863	−41.7235	0.0017	7231.7370	38.3561	–	–	–	23.10%
Keshmiri	9.8274	1.79 $\times$ 10^5^	2.5895	−1.9618	2256.6849	–	–	–	25.94%
Khansary	−7756.8036	−0.3088	0.0039	2.0840	0.0445	–	–	–	24.07%
Sodeifian	−37.9028	0.0078	1.9762	8.53 $\times$ 10^–5^	−3.21 $\times$ 10^–4^	1.9738	–	–	23.71%
Belghait	24.5312	−0.0835	1.61 $\times$ 10^–5^	$2.145\times$ 10^–4^	−0.0847	−4.90 $\times$ 10^–5^	−0.4875	137.3061	16.21%

**Figure 3:**
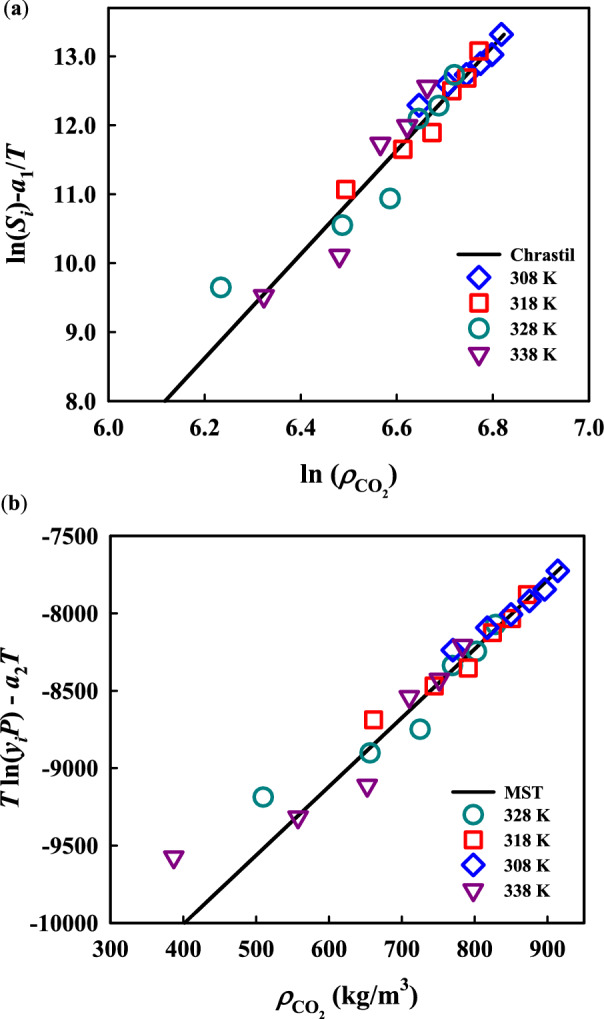
Self-consistency test for experimental data of palbociclib solubility in ScCO_2_ using (**a**) Chrastil and (**b**) MST models.

Due to several reasons such as difficulties related to the measurement of solubility values at small quantities, the reliability of the reported data to be used for process design, simulation and analysis should be confirmed dependently. For this purpose, thermodynamic consistency tests are of common methods to evaluate inherent inaccuracy of experimental data^45,46^. In this study, The simple and standard consistency tests using two semi-empirical models, i.e., Chrastil’s and MST models^47^ were conducted to confirm that the measured palbociclib solubility data were reliable. Figure 3 illustrates the consistency test results from the above mentioned models. As shown, the data points under various temperature conditions are approximately collapsed into a straight line, indicating that these data are self-consistent.

Generally, the dissolution of a solute molecule in ScCO_2_ is considered as a two-step process: vaporizing solute molecules from the solid state and dissolving solute molecules into the ScCO_2_ phase. The total and vaporization enthalpies of this two-step dissolution process can be estimated from the product of the semi-empirical model parameters and ideal gas constant *R* as follows^48,49^:

6 $${\Delta H}_{\mathrm{total}}=-{a}_{1}^{\mathrm{Chrastil}}\cdot R$$

7 $${\Delta H}_{\mathrm{vap}}=-{a}_{2}^{\mathrm{Bartle}}\cdot R$$

where ${a}_{1}^{\mathrm{Chrastil}}$ and ${a}_{2}^{\mathrm{Bartle}}$ are the regressed temperature coefficients of Chrastil’s and Bartle’s models, respectively. Once the total and vaporization enthalpies were obtained, the solvation enthalpy was calculated from

8 $${{\Delta H}_{\mathrm{sol}}=\Delta H}_{\mathrm{total}}-{\Delta H}_{\mathrm{vap}}$$

The total, vaporization and solvation enthalpies of palbociclib were determined to be 40.41 kJ/mole, 52.67 kJ/mole and −12.26 kJ/mole, respectively. These values can be meaningful in understanding the type and amount of energy needed for solid particles to dissolve in ScCO_2_. In this regard, a large value of total enthalpy may indicate that solute particles require more energy to dissolve in ScCO_2_ and release more heat when recrystallizing from ScCO_2_. Similarly, high value of vaporization enthalpy would show stronger intermolecular forces of solute against ScCO_2_ particles. However, these values are not measured experimentally but are determined based on assumptions made for developing the utilized semi-empirical models.

### Solubility correlation and prediction using cubic equation of state

Despite their simplicity, Peng–Robinson (PR)^50^ and Soave–Redlich–Kwong (SRK)^51^ equations of state are still of interest and have been shown to well correlate phase equilibrium data under high-pressure conditions for CO_2_ containing systems such as solid solute solubility in ScCO_2_^52,53^. These equations of state can apply to predict the phase equilibrium and solubility by combining them with a predictive liquid model^29,54–56^, such as UNIQUAC Functional-group Activity Coefficients (UNIFAC)^57^ or Conductor-like Screening Model-Segment Activity Coefficient (COSMOSAC)^58,59^. In this study, two correlative approaches based on PR EoS were used to model the palbociclib solubility data:

1. PR EoS using van der Waals mixing rules (PR + vdW),
2. PR EoS integrated with Wong-Sandler mixing rule^60,61^ and Wilson model^62^ (PR + WS + Wilson).

Besides, another predictive model based on PR EoS, Huron-Vidal mixing rule^63^ and COSMOSAC model (PR + HV + COSMOSAC), is applied to estimate the solubility of palbociclib in ScCO_2_. The molecular surface screening charges of palbociclib, key information for COSMOSAC, was obtained from the quantum mechanical and COSMO solvation calculations with the “b3lyp/6-31G(d.p)-cosmo” method^64^. Table 5 lists the equations of the above mentioned PR EoS based approaches.

**Table 5:** Investigated PR EoS based thermodynamic models.

PR EoS:	$P=\frac{RT}{\overline{V }-b(x)}-\frac{a(T,x)}{\overline{V }\left[\overline{V }+b(x)\right]+b\left[\overline{V }-b(x)\right]}$
PR + vdW^a^	$a=\sum_{i}\sum_{j}{{x}_{i}x}_{j}\sqrt{{a}_{i}{a}_{j}}(1-{k}_{ij})$ $b=\sum_{i}{x}_{i}{b}_{i}$
PR + WS + Wilson^b^	$\frac{a}{b}=\sum_{i}{x}_{i}\left(\frac{{a}_{i}}{{b}_{i}}\right)+\frac{{\overline{G} }^{E}}{{C}_{\mathrm{WS}}}$ $b=\frac{\sum_{i}\sum_{j}{{x}_{i}x}_{j}\left(\frac{{b}_{i}+{b}_{j}}{2}-\frac{\sqrt{{a}_{i}{a}_{j}}}{RT}\right)}{1-\sum_{i}{x}_{i}\left(\frac{{a}_{i}}{{b}_{i}RT}\right)-\frac{{\overline{G} }^{E}}{{C}_{\mathrm{WS}}RT}}$
PR + HV + COSMOSAC^c^	$\frac{a}{b}=\sum_{i}{x}_{i}\left(\frac{{a}_{i}}{{b}_{i}}\right)+\frac{{\overline{G} }^{E}}{{C}_{\mathrm{HV}}}$ $b=\sum_{i}{x}_{i}{b}_{i}$

*a_*i*_ and *b*_*i*_ are determined from critical temperature, critical pressure, and acentric factor of component *i* as described in the literature^50^.

^a^*k*_*ij*_ is an adjustable binary interaction parameter.

^b^ ${C}_{\mathrm{WS}}=\mathrm{ln}\left(\sqrt{2}-1\right)/\sqrt{2}$^60,61^. Excess Gibbs energy is determined from the Wilson model^62^: ${\overline{G} }^{E}=-RT\sum_{i}{x}_{i}\mathrm{ln}\left(\sum_{j}{x}_{j}{\Lambda }_{ij}\right)$ with ${\Lambda }_{ij}=\mathrm{exp}(-{u}_{ij}/RT)$ with ${u}_{ij}$ being adjustable binary interaction parameters.

^c^ ${C}_{\mathrm{HV}}=\mathrm{ln}\left(2\right)$^63^. Excess Gibbs energy ${\overline{G} }^{E}$ is calculated from the COSMOSAC model^58,59^ without any system-specific adjustable parameter.

The solid solute solubility in ScCO_2_ was determined by solving the equifugacity of palbociclib in supercritical fluid (SC) and its pure solid state (S)^65–67^:

9 $${f}_{\mathrm{pal}}^{\mathrm{SC}}\left(T, P,y\right)={f}_{\mathrm{pal}}^{\mathrm{S}}\left(T, P\right)$$

10 $$\mathrm{ln}\frac{{f}_{\mathrm{pal}}^{\mathrm{S}}\left(T, P\right)}{{f}_{\mathrm{pal}}^{\mathrm{L}}\left(T, P\right)}=\frac{{\Delta \overline{H} }_{m,\mathrm{pal}}}{R{T}_{m,\mathrm{pal}}}\left(1-\frac{{T}_{m,\mathrm{pal}}}{T}\right)+\frac{\left({\overline{V} }_{\mathrm{pal}}^{\mathrm{S}}-{\overline{V} }_{\mathrm{pal}}^{\mathrm{L}}\right)\left(P-{P}_{\mathrm{atm}}\right)}{RT}$$

where ${\Delta \overline{H} }_{m,\mathrm{pal}}$, ${T}_{m,\mathrm{pal}}$, ${\overline{V} }_{\mathrm{pal}}^{\mathrm{S}}$, ${\overline{V} }_{\mathrm{pal}}^{\mathrm{L}}$ and ${f}_{\mathrm{pal}}^{\mathrm{L}}$ are molar enthalpy of fusion, melting temperature, solid molar volume, liquid molar volume and the liquid phase fugacity of palbociclib, respectively. ${P}_{atm}$ as the atmospheric pressure was set to 101,325 Pa. The values of ${T}_{m,\mathrm{pal}}$ and ${\Delta \overline{H} }_{m,\mathrm{pal}}$ was taken from the literature^68^. The required values for ${\overline{V} }_{\mathrm{pal}}^{\mathrm{L}}$, ${f}_{\mathrm{pal}}^{\mathrm{L}}$ and ${\overline{f} }_{\mathrm{pal}}^{\mathrm{SC}}$ were determined from the studied EoS. In addition, the molecular cavity volume in the COSMO solvation calculation was used as the solid molar volume (${\overline{V} }_{\mathrm{pal}}^{\mathrm{S}}$).

Table 6 summarizes the optimal values of binary interaction parameters in PR + vdW and PR + WS + Wilson, which were obtained from regressing experimental palbociclib solubility, and the AARD% using these optimized parameters, which are 31.1% and 33.3% from PR + vdW and PR + WS + Wilson, respectively. It should be noted that the objective function for optimizing binary interaction parameters in PR + vdW and PR + WS + Wilson is ALD-*y* ($=\frac{1}{N}\sum_{i=1}^{N}\left|{{\mathrm{log}}_{10}y}_{i}^{\mathrm{exp}}-{{\mathrm{log}}_{10}y}_{i}^{\mathrm{cal}}\right|$, where ALD stands for average absolute logarithmic deviation). Such deviations are about twice those from Bian’s and Belghait’s models and slightly larger than those from the other eight semi-empirical models. Although PR EoS based approaches give slightly larger overall deviations, the obtained binary interaction parameter values could be used for phase behavior prediction of multicomponent systems containing palbociclib and CO_2_^69^. The AARD% from PR + HV + COSMOSAC is 79.6% (Table 6) and twice larger than the above two correlation approaches. However, it should be noted that this approach is the only approach without system-specific adjustable binary interaction parameters. Such an AARD% corresponds to 0.781 in terms of ALD-*y*. Such a deviation is similar to the overall ALD-*y* of using PSRK EoS and PR + COSMOSAC EoS in predicting solubility of 57 drug-like solid solutes^28,29^. This result demonstrates that combining PR EoS and COSMOSAC could at least provide a rough solubility estimation with sufficient data of the studied solid solute.

**Table 6:** Parameter values in PR EoS based thermodynamic models.

Model	*k*_12_ (-)	*u*_12_ (J/mol)	*u*_21_ (J/mol)	AARD%	ALD-y* (-)
PR + vdW	−3.783 $\times$ 10^–2^	–	–	31.1%	0.229
PR + WS + Wilson	–	1.660 $\times$ 10^3^	4.257 $\times$ 10^4^	33.3%	0.242
PR + HV + COSMOSAC	–	–	–	79.6%	0.781

* ALD-*y* $=\frac{1}{N}\sum_{i=1}^{N}\left|{{\mathrm{log}}_{10}y}_{i}^{\mathrm{exp}}-{{\mathrm{log}}_{10}y}_{i}^{\mathrm{cal}}\right|$ with *N* being the number of experimental data.

**Figure 4:**
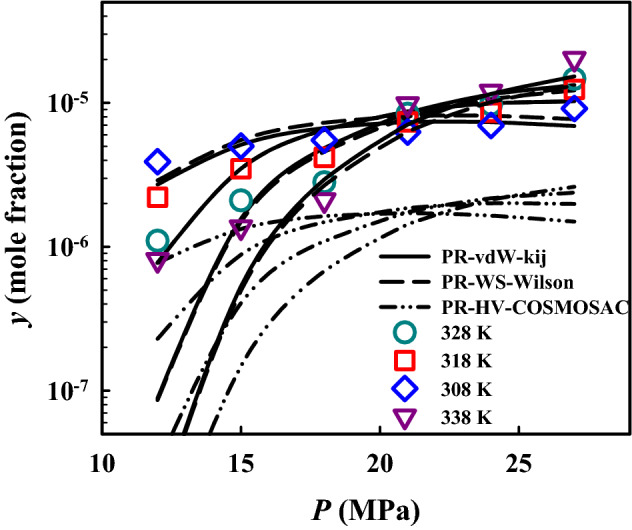
Palbociclib solubility in ScCO_2_ from experimentation and PR EoS based thermodynamic models.

Figure 4 compares the solubility of palbociclib in ScCO_2_ from the experiment and the PR EoS based thermodynamic models. In general, the solubility calculation results from the two correlative approaches are similar and agree with the experimental data. For the results from PR + HV + COSMOSAC, the solubility increasing trend due to increasing temperature or pressure is found to be similar to the above two correlative approaches, including the crossover region. However, it underestimates the solubility by nearly an order of magnitude. Since approaches based on combining PR EoS with the COSMOSAC model through a ${\overline{G} }^{E}$-based mixing rule have been demonstrated to reasonably predict binary vapor–liquid equilibria under high-pressure conditions for systems containing CO_2_, the studied predictive approach may be able to roughly estimate the palbociclib solubility in mixtures of ScCO_2_ with organic co-solvents.

## Conclusion

Reliable measurements of drug solubility in ScCO_2_, the most regarded solvent, provide the required information to design and develop the relevant processes in the pharmaceutical industry. In this work, experimental solubility of palbociclib, a cancer growth blocker, in ScCO_2_ was measured at temperatures between 308 and 338 K and pressures within 12–27 MPa. Afterwards, these experimental data were correlated by ten semi-empirical equations. The obtained results revealed that the Bian’s model had a closer fitting to the experimental data with the lowest AARD% compared to the other semi-empirical equations. Furthermore, the self-consistency of the palbociclib solubility data was proved by the studied semi-empirical equations. Besides, the capability of three integrated models based on Peng–Robinson EoS in describing the palbociclib solubility data was studied, among which PR + vdW showed better accuracy than the others. Finally, the total and vaporization enthalpies of palbociclib were found to be 40.41 and 52.67 kJ/mole, respectively.

## Data Availability

The datasets generated and/or analyzed during the current study are not publicly available due to confidential cases and are available from the corresponding author on reasonable request.

## References

[1] Sung H (2021). Global Cancer Statistics 2020: GLOBOCAN estimates of incidence and mortality worldwide for 36 cancers in 185 countries. CA A Cancer J. Clin..

[2] Cadoo KA, Gucalp A, Traina TA (2014). Palbociclib: an evidence-based review of its potential in the treatment of breast cancer. Breast Cancer (Dove Med Press).

[3] Yasir M, Asif M, Ashwani K, Aggarval A (2010). Biopharmaceutical classification system : An account. Int. J. PharmTech. Res..

[4] Türk M (2022). Particle synthesis by rapid expansion of supercritical solutions (RESS): Current state, further perspectives and needs. J. Aerosol Sci..

[5] Franco P, De Marco I (2020). Supercritical antisolvent process for pharmaceutical applications: A review. Processes.

[6] Razmimanesh F, Sodeifian G, Sajadian SA (2021). An investigation into Sunitinib malate nanoparticle production by US- RESOLV method: Effect of type of polymer on dissolution rate and particle size distribution. J. Supercrit. Fluids.

[7] Sodeifian G, Sajadian SA, Saadati Ardestani N (2016). Evaluation of the response surface and hybrid artificial neural network-genetic algorithm methodologies to determine extraction yield of Ferulago angulata through supercritical fluid. J. Taiwan Inst. Chem. Eng..

[8] Sodeifian G, Sajadian SA (2017). Investigation of essential oil extraction and antioxidant activity of Echinophora platyloba DC. Using supercritical carbon dioxide. J. Supercrit. Fluids.

[9] Sodeifian G, Sajadian SA, Honarvar B (2018). Mathematical modelling for extraction of oil from Dracocephalum kotschyi seeds in supercritical carbon dioxide. Nat. Prod. Res..

[10] Uwineza PA, Waśkiewicz A (2020). Recent advances in supercritical fluid extraction of natural bioactive compounds from natural plant materials. Molecules.

[11] Barbini S (2021). Multistage fractionation of pine bark by liquid and supercritical carbon dioxide. Biores. Technol..

[12] Sodeifian G, Razmimanesh F, Sajadian SA (2019). Solubility measurement of a chemotherapeutic agent (Imatinib mesylate) in supercritical carbon dioxide: Assessment of new empirical model. J. Supercrit. Fluids.

[13] Sodeifian G, Detakhsheshpour R, Sajadian SA (2019). Experimental study and thermodynamic modeling of Esomeprazole (proton-pump inhibitor drug for stomach acid reduction) solubility in supercritical carbon dioxide. J. Supercrit. Fluids.

[14] Jash A, Hatami T, Rizvi SSH (2020). Phosphatidylcholine solubility in supercritical carbon dioxide: Experimental data, thermodynamic modeling, and application in bioactive-encapsulated liposome synthesis. J. Supercrit. Fluids.

[15] Ardestani NS, Majd NY, Amani M (2020). Experimental measurement and thermodynamic modeling of capecitabine (an anticancer drug) solubility in supercritical carbon dioxide in a ternary system: Effect of different cosolvents. J. Chem. Eng. Data.

[16] Sodeifian G (2022). Solubility of prazosin hydrochloride (alpha blocker antihypertensive drug) in supercritical CO_2_: Experimental and thermodynamic modelling. J. Mol. Liq..

[17] Abadian M (2022). Experimental measurement and thermodynamic modeling of solubility of Riluzole drug (neuroprotective agent) in supercritical carbon dioxide. Fluid Phase Equilib..

[18] Sodeifian G, Sajadian SA, Daneshyan S (2018). Preparation of Aprepitant nanoparticles (efficient drug for coping with the effects of cancer treatment) by rapid expansion of supercritical solution with solid cosolvent (RESS-SC). J. Supercrit. Fluids.

[19] Sodeifian G, Sajadian SA (2018). Solubility measurement and preparation of nanoparticles of an anticancer drug (Letrozole) using rapid expansion of supercritical solutions with solid cosolvent (RESS-SC). J. Supercrit. Fluids.

[20] Sodeifian G, Sajadian SA (2022). Derakhsheshpour, R. CO_2_ utilization as a supercritical solvent and supercritical antisolvent in production of sertraline hydrochloride nanoparticles. J. CO2 Util..

[21] Crespi F (2017). Supercritical carbon dioxide cycles for power generation: A review. Appl. Energy.

[22] Di Maio E, Kiran E (2018). Foaming of polymers with supercritical fluids and perspectives on the current knowledge gaps and challenges. J. Supercrit. Fluids.

[23] Daneshyan S, Sodeifian G (2022). Synthesis of cyclic polystyrene in supercritical carbon dioxide green solvent. J. Supercrit. Fluids.

[24] Fathi M, Sodeifian G, Sajadian SA (2022). Experimental study of ketoconazole impregnation into polyvinyl pyrrolidone and hydroxyl propyl methyl cellulose using supercritical carbon dioxide: Process optimization. J. Supercrit. Fluids.

[25] Chen M-Y (2022). Supercritical carbon dioxide decellularized xenograft-3D CAD/CAM carved bone matrix personalized for human bone defect repair. Genes.

[26] Brunner G (2005). Supercritical fluids: Technology and application to food processing. J. Food Eng..

[27] Sodeifian G, Usefi MMB (2022). Solubility, extraction, and nanoparticles production in supercritical carbon dioxide: A mini-review. ChemBioEng Rev..

[28] Cai Z-Z (2020). First-principles prediction of solid solute solubility in supercritical carbon dioxide using PR+COSMOSAC EOS. Fluid Phase Equilib..

[29] Wang H-W, Hsieh C-M (2022). Prediction of solid solute solubility in supercritical carbon dioxide from PSRK EOS with only input of molecular structure. J. Supercrit. Fluids.

[30] Azim MM (2022). Estimating the solubility of salsalate in supercritical CO_2_ via PC-SAFT modeling using its experimental solubility data in organic solvents. J. Supercrit. Fluids.

[31] Belghait A (2018). Semi-empirical correlation of solid solute solubility in supercritical carbon dioxide: Comparative study and proposition of a novel density-based model. C. R. Chim..

[32] Zhu H (2021). Machine learning based simulation of an anti-cancer drug (busulfan) solubility in supercritical carbon dioxide: ANFIS model and experimental validation. J. Mol. Liq..

[33] Zhang Y, Xu X (2021). Machine learning bioactive compound solubilities in supercritical carbon dioxide. Chem. Phys..

[34] Sadeghi A (2022). Machine learning simulation of pharmaceutical solubility in supercritical carbon dioxide: Prediction and experimental validation for busulfan drug. Arab. J. Chem..

[35] Sodeifian G, Sajadian SA, Ardestani NS (2017). Determination of solubility of Aprepitant (an antiemetic drug for chemotherapy) in supercritical carbon dioxide: Empirical and thermodynamic models. J. Supercrit. Fluids.

[36] Sodeifian G (2016). Extraction of oil from Pistacia khinjuk using supercritical carbon dioxide: Experimental and modeling. J. Supercrit. Fluids.

[37] Sodeifian G (2019). Production of Loratadine drug nanoparticles using ultrasonic-assisted Rapid expansion of supercritical solution into aqueous solution (US-RESSAS). J. Supercrit. Fluids.

[38] Saadati Ardestani N, Sodeifian G, Sajadian SA (2020). Preparation of phthalocyanine green nano pigment using supercritical CO2 gas antisolvent (GAS): experimental and modeling. Heliyon.

[39] Sodeifian, G., et al. CO_2_ utilization for determining solubility of teriflunomide (immunomodulatory agent) in supercritical carbon dioxide: Experimental investigation and thermodynamic modeling. J. CO2 Util. **58**, 101931 (2022).

[40] Sodeifian G, Sajadian SA, Derakhsheshpour R (2019). Experimental measurement and thermodynamic modeling of Lansoprazole solubility in supercritical carbon dioxide: Application of SAFT-VR EoS. Fluid Phase Equilib..

[41] Zabihi S (2021). Measuring salsalate solubility in supercritical carbon dioxide: Experimental and thermodynamic modelling. J. Chem. Thermodyn..

[42] Pishnamazi M (2021). Chloroquine (antimalaria medication with anti SARS-CoV activity) solubility in supercritical carbon dioxide. J. Mol. Liq..

[43] Esfandiari N, Sajadian SA (2022). Experimental and modeling investigation of Glibenclamide solubility in supercritical carbon dioxide. Fluid Phase Equilib..

[44] Khudaida SH (2023). Solid solubility measurement of haloperidol in supercritical carbon dioxide and nanonization using the rapid expansion of supercritical solutions process. J. Supercrit. Fluids.

[45] Valderrama JO, Zavaleta J (2006). Thermodynamic consistency test for high pressure gas–solid solubility data of binary mixtures using genetic algorithms. J. Supercrit. Fluids.

[46] Valderrama JO, Faúndez CA, Campusano R (2019). An overview of a thermodynamic consistency test of phase equilibrium data. Application of the versatile VPT equation of state to check data of mixtures containing a gas solute and an ionic liquid solvent. J. Chem. Thermodyn..

[47] Méndez-Santiago J, Teja AS (1999). The solubility of solids in supercritical fluids. Fluid Phase Equilib..

[48] Chrastil J (1982). Solubility of solids and liquids in supercritical gases. J. Phys. Chem..

[49] Bartle KD (1991). Solubilities of solids and liquids of low volatility in supercritical carbon dioxide. J. Phys. Chem. Ref. Data.

[50] Peng D-Y, Robinson DB (1976). A new two-constant equation of state. Ind. Eng. Chem. Fundam..

[51] Soave G (1972). Equilibrium constants from a modified Redlich–Kwong equation of state. Chem. Eng. Sci..

[52] Hsieh C-M, Vrabec J (2015). Vapor–liquid equilibrium measurements of the binary mixtures CO_2_ + acetone and CO_2_ + pentanones. J. Supercrit. Fluids.

[53] Sodeifian G (2022). Measurement and modeling of metoclopramide hydrochloride (anti-emetic drug) solubility in supercritical carbon dioxide. Arab. J. Chem..

[54] Hsieh C-M, Windmann T, Vrabec J (2013). Vapor-liquid equilibria of CO_2_+C1-C5 alcohols from the experiment and the COSMO-SAC model. J. Chem. Eng. Data.

[55] Merker T (2013). Fluid-phase coexistence for the oxidation of CO_2_ expanded cyclohexane: Experiment, molecular simulation, and COSMO-SAC. AIChE J..

[56] Cai Z-Z, Hsieh C-M (2020). Prediction of solid solute solubility in supercritical carbon dioxide with and without organic cosolvents from PSRK EOS. J. Supercrit. Fluids.

[57] Fredenslund A, Jones RL, Prausnitz JM (1975). Group-contribution estimation of activity coefficients in nonideal liquid mixtures. AIChE J..

[58] Hsieh C-M, Sandler SI, Lin S-T (2010). Improvements of COSMO-SAC for vapor-liquid and liquid-liquid equilibrium predictions. Fluid Phase Equilib..

[59] Bell IH (2020). A benchmark open-source implementation of COSMO-SAC. J. Chem. Theory Comput..

[60] Wong DS-H, Sandler SI (1992). A theoretically correct mixing rule for cubic equations of state. AIChE J..

[61] Wong DS-H, Orbey H, Sandler SI (1992). Equation of state mixing rule for nonideal mixtures using available activity-coefficient model parameters and that allows extrapolation over large ranges of temperature and pressure. Ind. Eng. Chem. Res..

[62] Wilson GM (1964). Vapor-liquid equilibrium. 11. New expression for excess free energy of mixing. J. Am. Chem. Soc..

[63] Huron M-J, Vidal J (1979). New mixing rules in simple equations of state for representing vapor-liquid-equilibria of strongly non-ideal mixtures. Fluid Phase Equilib..

[64] Chen W-L (2016). A critical evaluation on the performance of COSMO-SAC models for vapor-liquid and liquid-liquid equilibrium predictions based on different quantum chemical calculations. Ind. Eng. Chem. Res..

[65] Kikic I, Lora M, Bertucco A (1997). A thermodynamic analysis of three-phase equilibria in binary and ternary systems for applications in rapid expansion of a supercritical solution (RESS), particles from gas-saturated solutions (PGSS), and supercritical antisolvent (SAS). Ind. Eng. Chem. Res..

[66] Ting Y-H, Hsieh C-M (2017). Prediction of solid solute solubility in supercritical carbon dioxide with organic cosolvents from the PR+COSMOSAC equation of state. Fluid Phase Equilib..

[67] Chen C-Y (2018). Prediction of solid-liquid-gas equilibrium for binary mixtures of carbon dioxide + organic compounds from approaches based on the COSMO-SAC model. J. Supercrit. Fluids.

[68] Liu W-D (2022). Thermodynamic modelling, Hansen solubility parameter and solvent effect of palbociclib in fourteen pure solvents at different temperatures. J. Mol. Liq..

[69] Li Q (2004). Modeling of the solubility of solid solutes in supercritical CO_2_ with and without cosolvent using solution theory. Kor. J. Chem. Eng..

[70] Hsieh C-M, Lin S-T (2008). Determination of cubic equation of state parameters for pure fluids from first principle solvation calculations. AIChE J..

[71] Liang H-H (2019). Improvement to PR+COSMOSAC EOS for predicting the vapor pressure of nonelectrolyte organic solids and liquids. Ind. Eng. Chem. Res..

[72] Lee BI, Kesler MG (1975). A generalized thermodynamic correlation based on three-parameter corresponding states. AIChE J..

[73] Kumar SK, Johnston KP (1988). Modelling the solubility of solids in supercritical fluids with density as the independent variable. J. Supercrit. Fluids.

[74] Bian X-Q (2016). A five-parameter empirical model for correlating the solubility of solid compounds in supercritical carbon dioxide. Fluid Phase Equilib..

[75] Garlapati C, Madras G (2010). New empirical expressions to correlate solubilities of solids in supercritical carbon dioxide. Thermochim. Acta.

[76] Keshmiri K, Vatanara A, Yamini Y (2014). Development and evaluation of a new semi-empirical model for correlation of drug solubility in supercritical CO_2_. Fluid Phase Equilib..

[77] Khansary M (2014). Representing solute solubility in supercritical carbon dioxide: A novel empirical model. Chem. Eng. Res. Des..

[78] Haghtalab A, Sodeifian G (2002). Determination of the Discrete Relaxation Spectrum for Polybutadiene and Polystyrene by a Non-linear Regression Method. Iran. Polym. J..

[79] Sodeifian G, Haghtalab A (2004). Discrete relaxation spectrum and K-BKZ constitutive equation for PVC, NBR and their blends. Appl. Rheol..

